# Club Foot*Read before the Buffalo Medical Association.

**Published:** 1883-11

**Authors:** Herman E. Hayd

**Affiliations:** M. D., M. R. C. S., Eng.


### BUFFALO

### Medical and Surgical Journal.

Vol. XXIII. NOVEMBER, 1883. No. 4.

### Original Communications.

### Club Foot.*

By Herman E. Hayd, M. D., M. R. C. S., Eng.

*Mr. Chairman and Gentlemen:*—Perhaps there is no special department in the vast domain of surgery that has made such progressive strides as orthopædia. Known slightly to the ancient surgeons, practiced by Hippocrates and his followers, to a very limited degree, and developed to its present scientific importance and practical utility during our own period, and largely during the last decade. It is two hundred years ago since tenotomy was first performed by a Dutch surgeon, for the relief of torticollis. For nearly one hundred years it fell into disuse, on account of the very erroneous ideas held by the most eminent surgeons regarding the extreme sensibility of tendons, and the very grave dangers attending their division. But with increased and advanced ideas in pathology, with the subject of inflammation and reparative union, well studied and well understood, with the assistance of anæsthetics and antiseptics, to encourage the surgeon in his experimental labors, this department soon had its ranks well filled with earnest workers, and

* Read before the Buffalo Medical Association.

thus, at this early day, Valentine Mott's prophetic visions have been realized, and orthopædic surgery, as if by magic touch, has unbound the fettered limbs, restored symmetry to the distorted form, given mobility to the imprisoned tongue, and directness to the orb of vision.

I may here briefly say, that amongst the great number of orthopædic surgeons in the world, with all their valuable contributions, we are mostly indebted to Little, in England, to Stromeyer, on the Continent, and Sayre, in America, for the great work which they individually have done.

Club foot may be divided into two great classes, from an etiological standpoint, namely, paralytic deformities and spastic or spasmodic distortions, which may be either congenital or acquired. And these, again, can be subdivided into various forms, according to the degree and character of the deformity. Thus we have talipes equinus, in which the heel is drawn up by the calf muscles, through their common tendon, the tendo achillis; talipes varus, in which the inner side of the foot is drawn upwards by the flexors, assisted by the tibials; talipes valgus, in which the outer side of the foot is drawn up by the peronei; talipes calcaneus, in which the foot is drawn up and the heel remains depressed. Again, various combinations of these occur, as talipes equino-varus, talipes equino-valgus, talipes calcan-varus, talipes calcan-valgus, etc., etc.

The cause of these deformities is often veiled in much obscurity, more especially those forms which occur "in utero." First of all, heredity seems to exercise some influence in this as in other deformities, and some very striking cases of hereditary transmission have occurred. For instance, Mr. Bryant says that the male branches of a family seem to be more prone than females in this respect, following the ordinary law of all deformities. Under his own care, he had a case of talipes varus, in which the child's father, grandfather and great grandfather, on the father's side, all had congenital talipes. Second, any condition, cause or disease, which will give rise to paralysis or loss of power to a single muscle, or a group of muscles, may give rise to talipes, by the opposing muscles becoming unduly contracted. Third, any irritative cause in the cerebro-spinal system, which will produce spasm of a muscle, perhaps at first overcome by the will, may be, in time, the cause of talipes. Under this latter cause, we may class many of the congenital varieties, for in these cases Little thinks there seems to be present some stimulus or irritant in the medulla spinalis, which acts upon certain ganglionic cells there, and thus keeps a certain muscle, or group of muscles, contracted, and in time produces a structural shortening of the member.

But this will not account for those cases in which there is not only a structural shortening of a muscle, but an ill-development of one or more bones, and possibly the whole foot, so that instead of having tarsal, metatarsal and phalangeal bones, the foot may be deprived of its digits, or metatarsals, and have nothing more than ill-formed tarsals covered with skin, etc. After having searched for a cause of these and like deformities of the hands, in works on general surgery, and to no avail, I resolved to go into this subject by referring to the physiological processes of development, and I now present to you a theory which will perhaps explain the pathology of congenital club foot and the more gross deformities of the feet and hands. After the sperm cell has united with the ovum, and gone through the process of segmentation, and its little fine granules and cells have arranged themselves to form the blastodermic layer, and its division into the epiblast and hypoblast has taken place, then I seek for the cause of these anomalies of development. Upon this lower layer or hypoblast certain cells become flattened, and as it were migrate to the centre and constitute a third layer or the mesoblast. From the epiblast is developed the epidermis, the cerebro-spinal system, the sensorial epithelium of the eye, ear, nose, etc. From the hypoblast, the epithelium of the gastro-intestinal tract, the abdominal organs and their ducts, etc., and from the mesoblast is developed the tissues and organs of the body, intervening between these two as the whole group of connective tissues, including the tissues proper, the muscles, bones, arteries, nerves, etc. Now, is it not possible that some cause, disease or injury on the part of the mother, or her conceived ovum, may interfere with the proper development of this mesoblastic layer, and consequently give rise to an improper development and formation of those tissues which nature destined them to form; and if that degeneration takes place in those cells which were to go on and develop the feet, a corresponding want of development in them would take place, and so also with the other members of the body.

While studying in University College Hospital, London, I had frequent opportunity of observing how often talipes varus, when of congenital origin, was associated with other anomalies in development, the most common being spina bivida. We also observed that as Mr. Heath cured this condition by injecting into the sac equal parts glycerine and iodine, the talipes was relieved, demonstrating that the talipes was the result of some irritative lesion in the spinal cord, and that as the pressure was relieved from the nerve roots, as they escaped through the vertebral foramina, the spasm was overcome.

The usual congenital form is talipes varus, and the more commonly acquired is talipes equinus or its modification, talipes equino-varus. It is this variety which I purpose inviting your attention to more particularly this evening. The accompanying photographs represent two little patients, the subjects of very marked talipes equino-varus, which Dr. Montgomery and myself operated upon some two months ago, and I have brought this little fellow here this evening to show you the result of the operation, and to demonstrate more easily the method of treatment adopted. His history is briefly as follows: Is eight years of age; family history good; always enjoyed good health since his early childhood; when thirteen months old had convulsions, which were probably the onset of infantile paralysis, as he was not enabled to walk for some months afterwards. Certain muscles were no doubt paralyzed and began to waste, others became unduly contracted, and thus the starting point of his deformity. But as in many of these cases, as time rolled on, all the muscles regained their muscular tonicity. Those over-contracted ones drew up the os calcis, as well as the scaphoid and the intermal cuneiform. He was therefore compelled to walk upon the side of his foot. Soon the astragalus was pushed out and became the heel; its ligamentous bands became relaxed. The external malleolus advanced forward until it was situated in front of the ankle joint, and nature, by a kind provision, developed a large cushion over the new heel, in the shape of a large bursa to protect her over-strained and over-worked joints; we thus found our little patient walking upon the outer borders of his feet, one leg crossing over and in front of the other, and a large bursa, the size of a hen's egg, situated over each astragalus and cuboid. On account of this distorted position the knee joints were strained, their ligaments became stretched, and so a partial internal dislocation of the knee joint complicated his pedal deformity.

Probably there is no disease that so frequently gives rise to talipes as infantile paralysis, and it is especially interesting to observe how certain groups of muscles, or certain individual muscles, as it were, are invidiously picked out and often become paralyzed.

As you are all aware, in the anterior coruna of the spinal cord are to be found a number of large nerve cells from which trophic fibres are given off; and in this peculiar affection (infantile paralysis) we find, upon microscopical examination, that these cells have degenerated, have become atrophied, and fibrous tissue has replaced them, the cord in different places has become sclerosed, and the greatest changes are to be seen in those portions of the cord whose function it was to preside over the muscles which have become paralyzed. So in certain irritative conditions affecting the antero-lateral columns, we get rigidity of certain muscles, as in spastic paralysis or bilateral sclerosis, and thus, on the one hand, from a certain pathological lesion, we get either a paralytic or a spastic distortion; paralytic if the nerve center be degenerated, and spastic if it be irritated.

It is very convenient, and at the same time practical, to divide talipes equino-varus into certain divisions according to the degree of the deformity; and I think Little's classification is a very good one; for instance, his first class is the slightest deformity, and in which the position of the front of the foot, when inverted, is such that the angle formed by it with the inside of the leg is greater than a right angle, and in which the contraction is so moderate that the toes can be easily brought to a straight line by the hand of the surgeon, and the heel can be depressed to a natural position; second, those cases in which the inversion of the foot and elevation of the heel is about the same or slightly greater than the former, but no reasonable effort will temporarily extinguish the contraction or deformity; third, those cases in which the contraction of the soft parts and the displacement of the hard parts reaches the highest degree, so that the inner margin of the foot is situated at an acute angle with the inside of the leg, and sometimes in contact with it. Our case corresponded to this third variety, and the angle formed by the inner side of the left leg with the great toe was about an acute one, while that of the right was not so marked. One is not prone to believe that a foot could be so easily straightened after having occupied this acquired position so long, for the articular ends of the bones are more or less worn off by the constant attrition they were subjected to, and new articulations and new joints have been made. But yet, our every-day experience teaches us that the changes in the bones are but slight, and that unless the case be of congenital origin where the bones are too often ill-formed and ill-developed; even in club foot of long standing much can be expected and promised by operative measures.

Before taking up the treatment of talipes, let us briefly consider how nature sets about in her reparative process. When a tendon, inclosed within its sheath, is divided subcutaneously, the divided ends separate for a variable distance, in an infant for about half an inch, and in an adult from one to two inches, depending upon the condition of the muscle fibre. After a period of repose, the sheath becomes softer, thicker and more vascular, its blood vessels are seen to be dilated, tortuous and varicose. About the divided ends of the sheath, a fibrinous blastema is thrown out, made up of an abundance of leucocythes and lymph cells, filled with nuclei and nucleoli, which soon extends and fills up the space between the separated ends. Little capillary blood vessels make their appearance in this mass. These lymph cells become gradually more and more elongated and fusiform in shape, until finally they become stretched out into thin, delicate, fibrous bands, which, in their turn, contract, press upon the little capillary loops, and change this once opaque, soft, gelatinous mass into firm, fibrous tissue. It is tough and resilient to the touch, and for years may be distinguished by its grayish, translucent color, from the white, glistening old tendon.

In a general way, the treatment of all foot deformities may be divided into the preventive and curative plans; and I am sure in no case is the old adage, "An ounce of prevention is worth more than a pound of cure," more applicable than in early club foot. If we meet with a case of congenital or early acquired club foot, we must give it our earliest attention, and we shall often find ourselves greatly remunerated for our trouble. By massage, electricity if the muscles be badly nourished, or certain ones unduly relaxed, daily working and manipulation of the foot, judicious stretching of the muscles so as to force the foot into its proper position, properly applied splints, and other appliances which the surgeon's own ingenuity will suggest, or well-directed strapping and bandaging, will save many a case from an operation. But after a careful trial of these several measures, we find that the shortening of the muscles, fasciæ, ligaments and integuments is not overcome; operate early, carefully and thoroughly, bearing in mind that all secondary operations avail but little, as adhesions, etc., are formed between the sheath and its tendon at various places, which interferes, yes, baffles our efforts for remedying the deformity. If an operation be decided upon, it must be done subcutaneously, and the tenotome which I have in my hand answers very well. The plan we adopted was, first, anæsthetize our patient, then put the tendons well upon the stretch by forcibly flexing or extending the foot, and at about a distance of a quarter of an inch from the sharp edge of the tendon, which was thrown out in bold relief, a small incision was made by the sharp-pointed knife through the skin. This being done, the blunt-pointed knife was passed well under the tendon, and, by a see-saw motion of the knife, the tendon being tense, its division was made known by that characteristic snap, and the knife carefully withdrawn. We divided, in both feet, the tendo achillis, the plantar fascia and the tibialis anticus, and with forcible extension overcame the contraction of the tibialis posticus. I think the cases are very few which require the division of the tibials, and at all events, I believe in leaving them until one tries what forcible extension can do, and failing in this, divide them at another sitting. After having divided the tendons, we placed the feet upon a straight splint along the inner side of the leg, leaving them for three days in about the same position they were in previous to the operation. This is the practice of most continental as well as English surgeons, and I believe it is to be preferred to that plan of treatment advocated by Sayre, who at once breaks down the foot as much as possible and then puts it up in the plaster Paris on the same day as the operation is performed. First of all, the external wound heals more quickly and much more kindly than if left gaping, a natural consequence if the parts be torn asunder. Second, the slight hemorrhage which occurs in every subcutaneous operation is lessened and the little blood which has exuded into the opening made by the operation is more easily absorbed. Third, there is less danger of inflammatory trouble following, as the parts are left nearly in apposition with one another, and at perfect rest.

On the third day we again put our little patient under chloroform, and forcibly brought the feet into as good a position as possible, and held them there by means of a plaster Paris bandage. In about ten days we took off this bandage, and once more forcibly manipulated the feet and got them into still better position, and again applied the plaster casing. In another ten days we removed this bandage and took the measurements for a Tieman shoe, sold by Lyman & Jeffrey, once more readjusted the bandage and kept the feet in good position until the shoes were sent us. Then we applied the shoes, and with very little difficulty the little fellow soon began to walk. You will observe that on account of the knee complication, which, gentlemen, is now our great trouble, we were compelled to extend the iron brace, with knee and hip joints upon it, up to the hip, and there fastened it to a hip band. These boots have done very well, but first of all they are unnecessarily heavy, awkward and cumbersome, and shamefully expensive, the latter being a very unfortunate circumstance, as most children affected with club foot deformities, at all events to such a degree, are the offspring of very poor people.

In those very marked paralytic cases, where the leg may be thrown about like a flail and the ligaments of the knee and ankle joints greatly relaxed, little can be done. Still, friction, shampooing, electricity, salt water baths and douches, in fact any agent which will tend to keep the limb as well nourished as possible, are the measures indicated.

In closing, gentlemen, permit me to say that after having seen a great many cases of club foot, both in hospital and private practice, in which operations were performed, a few very practical lessons have been taught me: First of all, we must be patient, exceedingly kind and gentle in all our actions, in order to enjoy the continued confidence of the parents or guardians of the little one whom we are inflicting so much pain upon. Second, let us rather over-estimate the amount of pain which must be endured and the amount of attention and physical labor which must be given by the attendants, or in the very midst of a brilliant success, when nothing remains but to keep the foot in position, our efforts will be thwarted, as mine were in the case of that pretty little girl whose photograph is being passed around, because so great a deformity usually occurs in the families of poor, unintelligent and sickly sentimental people, who would rather see their child deformed than see it suffer pain. Third, if possible operate in a hospital or some such institution where the immediate care of the child is left to suitable and capable nurses.

